# The Use of a Pressure-Indicating Film to Determine the Effect of Liner Type on the Measured Teat Load Caused by a Collapsing Liner

**DOI:** 10.3390/s17040855

**Published:** 2017-04-13

**Authors:** Susanne Demba, Viktoria Paul, Christian Ammon, Sandra Rose-Meierhöfer

**Affiliations:** 1 Leibniz-Institute for Agricultural Engineering and Bioeconomy e.V. (ATB), Department of Engineering for Livestock Management, Max-Eyth-Allee 100, Potsdam 14469, Germany; VPaul@atb-potsdam.de (V.P.); cammon@atb-potsdam.de (C.A.); 2 Hochschule Neubrandenburg, University of Applied Sciences, Department of Agricultural Machinery, Brodaer Straße 2, Neubrandenburg 17033, Germany; rose@hs-nb.de

**Keywords:** sensor-based detection, pressure sensor, teat load, liner collapse, machine milking

## Abstract

During milking the teat cup liner is the interface between the teat of a dairy cow and the milking system, so it should be very well adapted to the teat. Therefore, the aim of the present study was to determine the effect of liner type on the directly measuring teat load caused by a collapsing liner with a pressure-indicating film. The Extreme Low pressure-indicating film was used to detect the effect of six different liners on teat load. For each liner, six positions in the teat cup were specified, and six repetitions were performed for each position with a new piece of film each time. Analysis of variance was performed to detect differences between the six liners, the positions within a liner, and the measuring areas. The pressure applied to the teat by a liner depends on the technical characteristics of the liner, especially the shape of the barrel, and for all tested liners, a higher teat load was found at the teat end. In conclusion, with the help of pressure-indicating film, it is possible to determine the different effects of liner type by directly measuring teat load due to liner collapse.

## 1. Introduction

During machine milking, the teat cup liner is the interface between the teat of a dairy cow and the milking system; it transfers the force created by the pressure difference between the pulsation chamber and the interior of the liner directly to the teat tissue [1]. While the teat of a dairy cow is robustly constructed and well adapted to shear stress [2], machine milking can worsen the condition of the teat and teat tissue [3,4,5]. Therefore, it is important that the liner is very well adapted to the teat.

The most commonly used method to detect the impact of liner type is to visually evaluate teat condition based on teat color, swelling, ring formation at the teat base, and teat-end hyperkeratosis [3,6,7,8,9,10].

In addition to visually observing teat condition, sensor-based determination of the influence of liner type on the teat load caused by liner collapse can be performed with several measuring devices. Paulrud et al. [11] used infrared thermography as well as ultrasonography to monitor the influence of liner type on teat temperature and teat traits such as the teat cistern wall, teat cistern diameter, and teat canal length. The ultrasonography was also used by Gleeson et al. [12] and Gleeson, O’Callaghan, Meaney and Rath [8] to investigate differences in teat traits caused by different liners. Several studies have attempted to detect the influence of liner type on the teat load with the help of different pressure-sensitive sensors. Davis et al. [13] measured the compressive load applied to the teat by the closed liner using an artificial teat equipped with a miniature load cell and found that the compressive load of a liner is proportional to the thickness of the liner wall; the authors determined a curvilinear relationship between the insertion depth and the compressive teat load as well. Tol et al. [14] investigated the teat-liner interface using a flexible pressure-sensitive layer and found that conventional round liners concentrated the load over two sides of the end of the teat. In contrast, liners with softer material, reduced tension, a smaller barrel, and reduced mouthpiece depth distributed the pressure over a larger area of the teat, but the maximum pressure was always exerted at the teat end. Leonardi et al. [15] used an artificial teat sensor adapted from Davis, Reinemann and Mein [13] to estimate liner compression, and the round liner compression was positively correlated (R^2^ = 0.97 − 0.91) with the pressure difference through the liner wall. According to these authors, this sensor is only useful for round liners.

As the methods commonly used to detect the effect of liner type on the bovine teat are very subjective and because the tested sensor-based methods are very complex to use or have shown limited usability, the aim of this study was to determine the effectiveness of a pressure-indicating film in detecting the effect of liner type on the directly measuring teat load caused by a collapsing liner.

## 2. Materials and Methods

### 2.1. Experimental Setup

The experiment was carried out in the laboratory milking parlor of the Leibniz-Institute for Agricultural Engineering and Bioeconomy e.V. (ATB). A conventional milking cluster (GEA Group AG, Düsseldorf, Germany) was used, and the machine vacuum was adjusted to 40 kPa. Alternate pulsation was used at a rate of 60 min^−1^ and a 60:40 pulsation ratio.

### 2.2. Artificial Teat

An artificial teat made of silicone was used to investigate the influence of liner type on the teat load caused by a collapsing liner. The teat had a length and a mean diameter of 56 mm and 21 mm, respectively, and it was hollow with a teat wall thickness of 4.5 mm. According to the manufacturer, the silicone rubber had a Shore A hardness of 25, a density of 1.16 g·cm^−3^ at a temperature of 23 °C, a tensile strength of 5.00 N·mm^−2^, an ultimate elongation of 350%, a tear resistance of more than 20 N·mm^−1^, and a linear shrinkage of 0.5%.

### 2.3. Teat Cup Liners

The teat load on the artificial teat caused by liner collapse was measured for six different liners: a round silicone liner (SilRou), a round rubber liner with head ventilation (RubRouHV), a triangular rubber liner (RubTri), a concave rubber liner (RubCon), a round rubber liner (RubRou), and a square rubber liner (RubSqu). Table 1 shows the technical specifications of the tested liners.

### 2.4. Pressure-Indicating Film

The Prescale pressure-indicating films by Fujifilm (KAGER Industrieprodukte GmbH, Dietzenbach, Germany) are used to measure pressure, pressure distribution, and pressure balance and are available in mono- and two-sheet types. The two-sheet film consists of a color-forming layer and a color-developing layer, and when pressure is applied to the film, the microcapsules are broken so that the color-forming material reacts with the color-developing material. As a result, red patches appear on the film, and the density of the red color indicates several levels of pressure between 0.05 MPa and 50 MPa. Following the measuring procedure, the C-films can be visualized with a scanner (Epson Perfection V37/V370 Photo, KAGER Industrieprodukte GmbH) and analyzed with the FPD-8010E software by Fujifilm (KAGER Industrieprodukte GmbH). This software analyze six parameters: the proportion within the pressure-detection range of the film (effective rate, ER, in %), the surface area over which the color was generated (pressed area, PA, in mm^2^), the mean pressure on the area where the color was generated (average pressure, AP, in MPa), the maximum pressure on the area where the color was generated (maximum pressure, MP, in MPa), the product of the pressurization surface area and the average pressure (load, L, in N), and the measured area (MA, in mm^2^).

### 2.5. Data Collection

**Figure 1:**
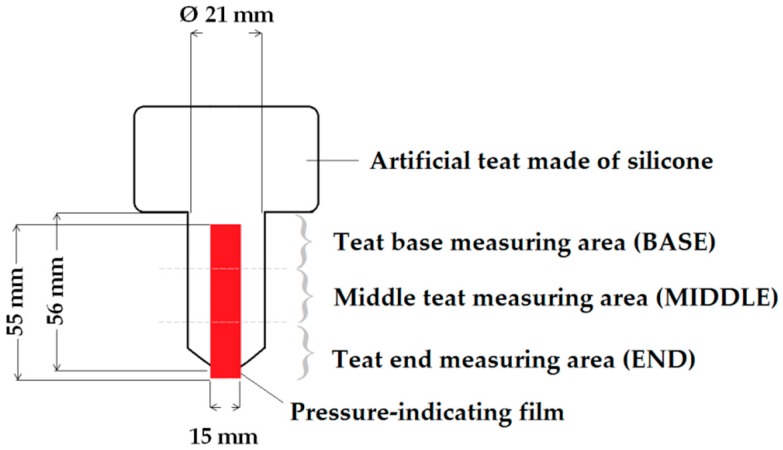
Schematic of the artificial teat with the pressure-indicating film and the three measuring areas.

The Extreme Low film (Prescale by Fujifilm; KAGER Industrieprodukte GmbH, Dietzenbach, Germany) was used to determine the influence of different liners on the measured teat load due to liner collapse. The pressure-indicating film was cut into pieces (15 mm × 55 mm), all of which were attached to the teat with tape (Figure 1).

**Figure 2:**
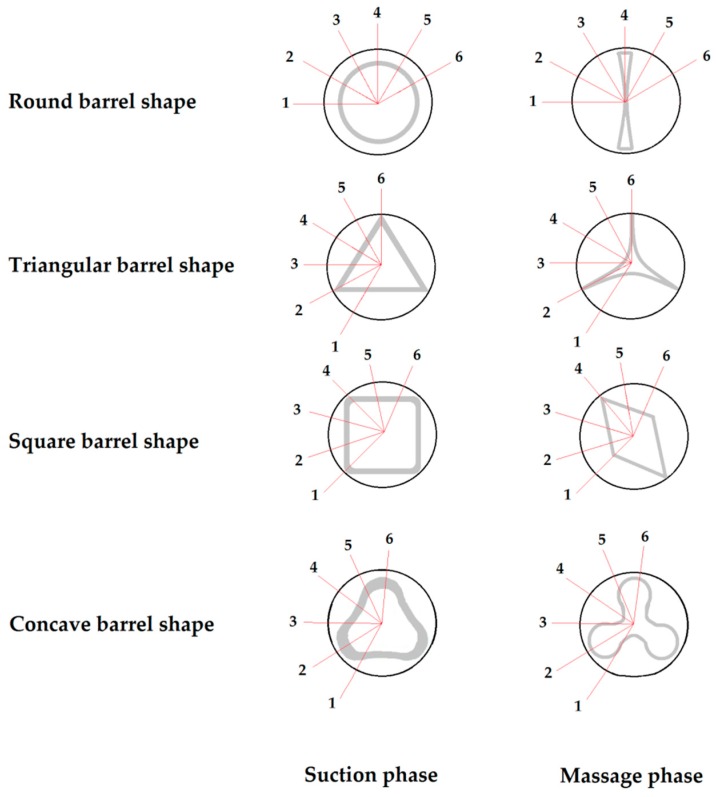
The measurement positions in 30° intervals on the teat cup of the artificial teat for each barrel shape during the suction and massage phases of the milking process.

Due to the small size of the sensor, the teat-sensor-combination was rotated five times in intervals of 30° to record the teat load at each position (Figure 2), and six repetitions were performed for each position. The artificial teat was inserted in the teat cup, and the liner was opened and closed for 30 s. After each session, the artificial teat was turned 30°, and the next measurement was recorded. Thus, the pressure-indicating film measured the pressure at each point of the liner surface at and between the chosen positions.

After measuring, the films were analyzed with the FDP-8010E software by Fujifilm (Prescale by Fujifilm; KAGER Industrieprodukte GmbH, Dietzenbach, Germany). The load, which is the product of the pressurized surface area and the average pressure (L in N), and the maximum pressure in the area over which color was generated (MP in MPa) were used to analyze the influence of the different liners. L and MP were calculated for the film at the three measuring areas (Figure 1). Therefore, the scanned film was divided into the teat base area (BASE), the middle teat area (MIDDLE), and the teat end area (END).

### 2.6. Statistical Analysis

Data were analyzed using the SAS 9.4 software package (SAS Institute Inc., Cary, NC, USA), and analysis of variance (ANOVA) was used to estimate the differences in L among the liners and the positions of the artificial teat in the teat cup within a liner for the whole measuring area using the MIXED procedure. The null hypothesis for L was that there were no differences in the tested trait between the liners and the positions within a liner. The following model was used to calculate the influence of the different liner types on L:

y_ijl_ = µ + L_i_ + P_j_ + (LP)_ij_ + ε_ijl_ (1)

where y_ijl_ is the observed value of the i-th liner (i = 1,…,6) and the j-th position of the artificial teat in the teat cup (j = 1,…,6) and the l-th repetition (l = 1,…,6); µ is the overall mean; L_i_ is the fixed effect of the liner type; P_j_ is the fixed effect of the position in the teat cup; (LP)_ij_ is the interaction between the liner and the position; and ε_ijl_ is the residual.

ANOVA was also used to estimate the differences in L among the liners, the position of the artificial teat in the teat cup, and the three measuring areas using the MIXED procedure, and the null hypothesis for L was that there were no differences among the liners, the positions, and the measuring areas of the tested trait. The following model was used to calculate the influence of the different liner types on L:

y_ijkl_ = µ + L_i_ + P_j_ + A_k_ + (LP)_ij_ + (LA)_ik_ + (PA)_jk_ + (LPA)_ijk_ + ε_ijkl_ (2)

where y_ijkl_ is the observed value of the i-th liner (i = 1,…,6), the j-th position of the artificial teat in the teat cup (j = 1,…,6), the k-th measuring area (k = BASE, MIDDLE, END), and the l-th repetition (l = 1,…,6); µ is the overall mean; L_i_ is the fixed effect of the liner type; P_j_ is the fixed effect of the position in the teat cup; A_k_ is the fixed effect of the measuring area; (LP)_ij_ is the interaction between the liner and the teat position; (PA)_jk_ is the interaction between the position and the measuring area; (LPA)_ijk_ is the interaction between the liner, the position and the measuring area; and ε_ijkl_ is the residual.

The GLIMMIX procedure was used to examine the differences in MP between the liners and the positions of the artificial teat in the teat cup within a liner for the whole measuring area, and the null hypothesis for MP was that there were no differences between the liners and the positions within a liner. A binomial distribution was assumed for MP, and the following model was used:

(3) $$P\left(y_{\mathrm{ijl}}\right)=\frac{e^{\eta }}{1+e^{\eta }}$$

The linear predictor η is calculated as follows:

η_ijl_ = μ + L_i_ + P_j_ + ε_ijl_ (4)

where µ is the overall mean; L_i_ is the fixed effect of the i-th liner type (i = 1,…,6); P_j_ is the fixed effect of the j-th position of the artificial teat in the teat cup (j = 1,…,6); and ε_ijl_ is the residual.

The GLIMMIX procedure was also used to examine the differences in MP between the liners, the position of the artificial teat in the teat cup, and the three measuring areas, and the null hypothesis for MP was that there were no differences between the liners, the positions, and the measuring areas. A binomial distribution was assumed for MP, and the following model was used:

(5) $$P\left(y_{\mathrm{ijkl}}\right)=\frac{e^{\eta }}{1+e^{\eta }}$$

The linear predictor η is calculated as follows:

η_ijkl_ = μ + L_i_ + P_j_ + A_k_ + ε_ijkl_ (6)

where µ is the overall mean; L_i_ is the fixed effect of the i-th liner type (i = 1,…,6); P_j_ is the fixed effect of the j-th position of the artificial teat in the teat cup (j = 1,…,6); A_k_ is the fixed effect of the k-th measuring area (k = BASE, MIDDLE, END); and ε_ijkl_ is the residual.

All tests were carried out at a significance level of 0.05.

## 3. Results

### 3.1. Load

#### 3.1.1. Differences between Positions within a Liner for the Whole Measuring Area and between the Measuring Areas within a Liner and Position

In a first step, the differences between the positions within a liner for the whole measuring area were determined. The results of the analysis of variance showed a significant influence of the liner (*p* < 0.0001), the position of the artificial teat in the teat cup (*p* = 0.0428), and the interaction between the liner and the position within a liner (*p* < 0.0001) on L.

For SilRou, L was at least 20.83 N higher at the position where the liner pressed the teat (Position 1) compared to the other positions, and with RubTri, L was 21.17 N higher at the position in the corner of the liner (Position 6) compared to where the liner pressed the teat (Position 4). Where the liner pressed the teat (Position 1), L was at least 21.67 N lower compared to the other positions (Position 2, 3, 5, 6) in RubRou, and for RubSqu, L was at least 22.33 N lower where the liner pressed the teat (Position 1) compared to the other positions. No differences were found between the positions with RubRouHV and RubCon.The standard error for all estimated means was 7.27 N.

**Figure 3:**
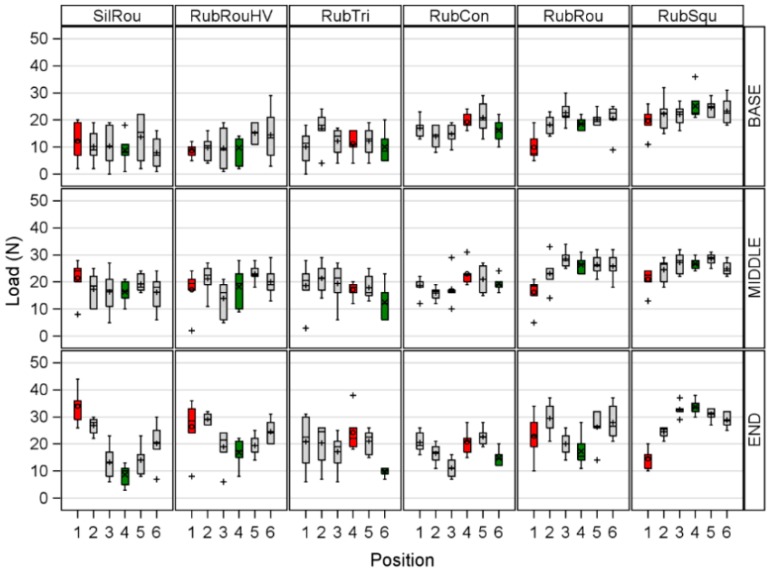
The differences in the measured Load between a round silicone liner (SilRou), a round rubber liner with head ventilation (RubRouHV), a triangular rubber liner (RubTri), a concave rubber liner (RubCon), a round rubber liner (RubRou), and a square rubber liner (RubSqu) depending on the position of the artificial teat in the teat cup (red and green boxes indicate the position where the liner compressed the teat and the position where it bended in the edges, respectively) and the measuring area with BASE = the teat base measuring area, MIDDLE = the middle teat measuring area, and END = the teat end measuring area.

In a second step, the measuring areas within a liner and a position were compared. Figure 3 shows the differences in L between the tested liners depending on the position of the artificial teat in the teat cup and the measuring area.

The analysis of variance showed a significant influence of the liner (*p* < 0.0001), the position of the teat in the liner (*p* = 0.0007), and the measuring area (*p* < 0.0001) on L. The interactions between the position and the liner (*p* < 0.0001), the liner and the measuring area (*p* < 0.0001), and the position and the measuring area (*p* = 0.0012) as well as the triple interaction between the position, the liner, and the measuring area (*p* < 0.0001) significantly influence L as well. Within all liners, the highest L was measured at the END compared to the BASE and MIDDLE. Table 2 shows the comparisons of the combinations of measuring areas per liner and the position of the artificial teat in the teat cup, all of which differ significantly.

No differences could be found between the measuring areas of RubCon, so it evenly distributed the pressure on the teat.

#### 3.1.2. Differences between the Liners

To determine differences between the tested liners the positions where the liners compressed the teat (COMP) and the position where the liners did not compress the teat (CORN) were compared between the liners. COMP included position 1 of SilRou, RubRouHV, RubRou, and RubSqu, and position 4 of RubTri and RubCon; CORN included the position 4 of SilRou, RubRouHV, RubRou, and RubSqu, and position 6 of RubTri and RubCon. The comparison of COMP at the BASE area showed that the applied L by RubSqu was 11.00 N, 9.67 N, and 9.83 N higher than this of RubRouHV, RubTri and RubRou, respectively. The L values of RubCon were 10.67 N and 9.33 N higher than these of RubRouHV and RubTri, respectively. The L values of SilRou were 9.83 N, 13.33 N, 11.17 N, and 19.50 N higher at the END than these of RubTri, RubCon, RubRou, and RubSqu, respectively. The applied L of RubRouHV was 11.83 N higher than this of RubSqu. No differences between the liners were found at the MIDDLE.

The comparison of CORN at the BASE resulted in a 9.67 N and 16.33 N lower L for SilRou compared to RubRou, and RubSqu, respectively. L of RubRouHV was 9.67 N, 8.83 N, and 15.50 N lower than of RubCon, RubRou, and RubSqu. The comparison of the three angular liners resulted in a 10.67 N higher load of RubSqu compared to RubTri and RubCon. At the MIDDLE L of RubRou and RubSqu was 10.00 N and 10.33 N higher than this of SilRou, respectively. The L values of RubSqu were 25.00 N, 16.83 N, 9.17 N, 9.17 N, and 16.33 N higher at the END compared to SilRou, RubRouHV, RubTri, RubCon, and RubRou, respectively.

The standard error for all estimated means was 3.10 N.

### 3.2. Maximum Pressure

#### 3.2.1. Differences between the Positions within a Liner for the whole Measuring Area

No significant differences between the tested liners and the position of the artificial teat in the teat cup within a liner in MP could be found.

#### 3.2.2. Differences between Liners, Positions, and Measuring Areas

**Figure 4:**
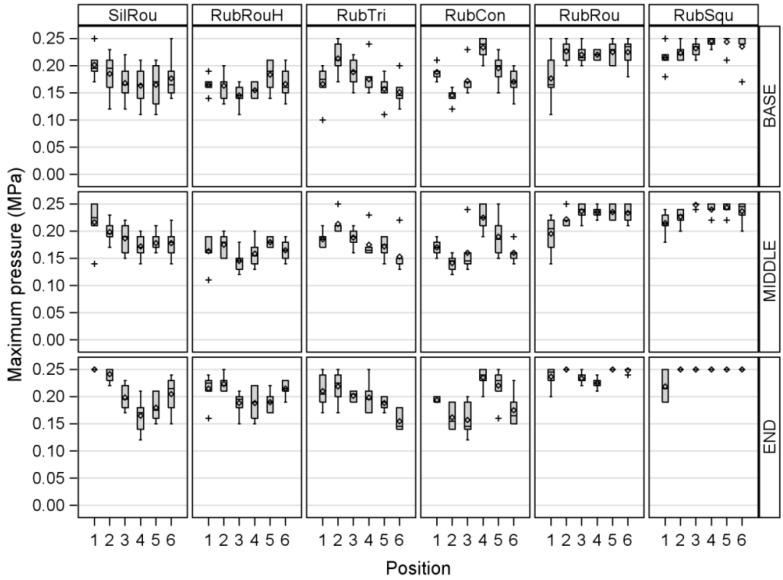
The differences in the measured Maximum pressure between a round silicone liner (SilRou), a round rubber liner with head ventilation (RubRouHV), a triangular rubber liner (RubTri), a concave rubber liner (RubCon), a round rubber liner (RubRou), and a square rubber liner (RubSqu) depending on the position of the artificial teat in the teat cup and the measuring area with BASE = the teat base measuring area, MIDDLE = the middle teat measuring area, and END = the teat end measuring area.

Figure 4 shows the differences in MP between the tested liners depending on the position of the artificial teat in the teat cup and the measuring area.

The analysis of variance showed a significant influence of the liner (*p* < 0.0001) and the measuring area (*p* = 0.0406) on MP, but no significant effect of the position of the teat in the teat cup on MP could be found. The MP values were higher for RubRou and RubSqu compared to the other tested liners; the MP of RubRou was 0.04–0.05 MPa higher than that of SilRou, RubRouHV, RubTri, and RubCon. RubSqu showed the highest MP values; the applied pressure was 0.05–0.06 MPa higher than that of SilRou, RubRouHV, RubTri, and RubCon. The MP values were 0.02 MPa higher at the END compared to the BASE, but no significant differences in MP could be found among the other measuring areas.

### 3.3. Conspicuousness Effect of RubCon

**Figure 5:**
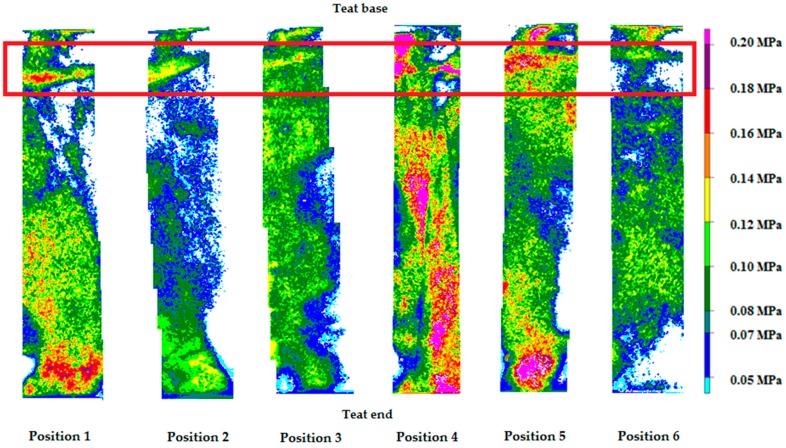
Extreme Low Prescale film scans of each measured position of the artificial teat in the teat cup for the concave liner with the teat base at the top of the figure and the teat end below.

The RubCon measurements exhibited a ring at the teat base where more pressure was applied by the liner. This ring was generally apparent within every measurement independent of the position of the artificial teat in the teat cup (Figure 5).

## 4. Discussion

Different liner types significantly influenced the values of both L and MP, and the different positions of the artificial teat in the teat cup within a liner also affected the teat load caused by a collapsing liner. For SilRou, L was highest at the position where the liner pressed the teat and lowest where the liner bends at the edges (Position 4); these results were confirmed by Tol, Schrader and Aernouts [14] who found similar effects. In contrast, the results for RubRou in the present study did not agree with those of Tol, Schrader and Aernouts [14]. For RubTri, L was higher at the corner than at the position where the liner pressed the teat, which is inconsistent with the results of Tol, Schrader and Aernouts [14] who found only three pressure spots (the three sides where the liner touched the teat) within a triangular liner. However, their artificial teat was 20 mm longer, had a tapered shape, and a 2.5 mm-thinner teat wall compared to the artificial teat used in the present investigation, so this could explain the different results.

Teat load increased from the BASE through the MIDDLE to the END during liner collapse. This result partially agrees with Tol, Schrader and Aernouts [14] who found a similar pressure distribution in round liners. In the present study, the highest teat load was found at the END compared to the other measuring areas. Muthukumarappan et al. [16] confirmed these results, finding that the maximum pressure was applied within 1 or 2 mm of the teat end, and Tol, Schrader and Aernouts [14] also found that the maximum pressure was always applied to the teat end.

The comparison of the different liner types resulted in the highest teat load for SilRou at COMP and END, which disagrees with Tol, Schrader and Aernouts [14]. They found the highest pressure values for a round-square and a triangular liner. Furthermore, the angular liners had a higher teat load at CORN compared with the round liners, which disagrees with the results of Tol, Schrader and Aernouts [14] as well. They found no load in the corners of a triangular liner and observed pressure all around the teat with a square liner. In contrast, Zucali et al. [17] found higher incidences of teat-end hyperkeratosis on farms milking with triangular liners. The use of triangular rubber liners, compared to round rubber liners, did not reduce the traumatization of teats due to milking at different machine vacuum levels [18]. Schukken et al. [19] found a lower frequency of teat ends with crack and teat-end hyperkeratosis in teats milked with square liners. At COMP, the teat load caused by SilRou was higher than that by RubRou, which led to the assumption that a softer material resulted in a higher teat load. Paulrud, Clausen, Andersen and Rasmussen [11] found that milking with a liner made of softer material resulted in colder teats after milking, but Tol, Schrader and Aernouts [14] found similar pressure values for liners made of silicone and rubber. The observations regarding liner material in the present study could be explained by the higher SilRou Touch Point values. The results of Davis, Reinemann and Mein [13] that the compressive load of a liner is proportional to the thickness of the liner wall can neither be confirmed nor refuted because the majority of the liners tested in the present study had a wall thickness of 2.0 mm.

RubCon showed a pressurization ring at the BASE area in all tested positions, which could be explained by the 3 mm-smaller mouthpiece bore diameter. Haeussermann, Britten, Britten, Pahl, Älveby and Hartung [10] compared a concave and a round liner in terms of the effect of each on the degree and roughness of teat-end hyperkeratosis, and they found a lower incidence of rough teat-end hyperkeratosis with RubCon. Unfortunately, they did not investigate the influence of the concave liner on the ring formation at the teat base, so its effects on the teat base remain unknown. On the other hand, Gleeson, O’Callaghan, Meaney and Rath [8] found no significant differences in teat condition between wide-bored and narrow-bored liners.

The measurements with the pressure-indicating film were not influenced by machine vacuum, sensor bending, and shear force. According to Demba et al. [20] neither negative pressure nor sensor bending influenced the measurements by pressure-indicating film. These films do not support shear stress as well [21].

## 5. Conclusions

It can be concluded that the pressure-indicating film can be used to determine the influence of different liner types on the teat load caused by a collapsing liner because it directly measures the pressure and the load due to liner collapse. Using these measured pressure values, it is possible to objectively compare the effect of the liners on the teat. The pressure applied to the teat depends on the technical characteristics of the teat cup liner, especially the barrel shape. However, there is still a lack of information about the load applied by a collapsing liner, which is necessary to massage the teat, so further studies are needed. Furthermore, it is important to determine the dimensions of the teats in a dairy herd to select the best adapted liner. The artificial teat used in the present investigation was flexible, but in further studies, a teat that is more similar to a natural teat will be used.

## Figures and Tables

**Table 1:** Overview of the tested teat cup liners and their different characteristics.

Liner	1	2	3	4	5	6
Abbreviation	SilRou	RubRouHV	RubTri	RubCon	RubRou	RubSqu
Material	silicone	rubber	rubber	rubber	rubber	rubber
Mouthpiece bore diameter (mm)	23	23	23	20	23	23
Barrel shape	round •	round •	triangular ∆	concave ∆	round •	square □
Barrel diameter at 75 mm (mm)	25	23	-	-	24	-
Side edge length (a) at 75 mm (mm)	-	-	30	30	-	25
Inscribed circle ^1^ at 75 mm (mm)	-	-	8.7	8.7	-	7.2
Circumradius ^2^ at 75 mm (mm)	-	-	17.3	17.3	-	14.4
Liner length (mm)	169	156	150	149	159	151
Touchpoint (kPa)	18.4	15.1	-	-	12.9	-
Wall thickness (mm)	2.0	3.0	2.0	2.0	2.0	2.0
Head ventilation	no	yes	yes	no	no	no

^1^ Inscribed circle = √3/6 × a; ^2^ Circumradius = √3/3 × a.

**Table 2:** The significant differences in L (measured load) and 95%-confidence intervals (CI) between the measuring areas (BASE = teat base measuring area, MIDDLE = teat middle measuring area, END = teat end measuring area) for each liner and position of the artificial teat in the teat cup with a standard error of 3.10 N.

Liner	Position	Compared Measuring Areas	Difference in L (N)	CI	
				Lower	Upper
1	1	BASE-MIDDLE	−9.33	−18.50	−0.17
1	1	BASE-END	−21.83	−33.97	−9.70
1	1	MIDDLE-END	−12.50	−24.64	−0.36
1	2	BASE-END	−16.67	−25.83	−7.50
1	2	MIDDLE-END	−9.50	−18.67	−0.33
1	6	BASE-END	−12.33	−21.50	−3.17
2	1	BASE-END	−17.67	−26.90	−8.43
2	2	BASE-MIDDLE	−11.50	−20.74	−2.26
2	2	BASE-END	−19.50	−28.74	−10.26
2	3	BASE-END	−9.50	−18.74	−0.26
2	6	BASE-END	−10.00	−19.24	−0.76
3	1	BASE-END	−10.83	−20.03	−1.64
3	4	BASE-END	−12.83	−22.03	−3.64
5	1	BASE-END	−13.00	−22.15	−3.85
5	2	BASE-END	−11.33	−20.48	−2.18
6	3	BASE-END	−10.67	−19.87	−1.46
